# Advances in ERECTA Family Regulation of Female Gametophyte Development in *Arabidopsis thaliana*

**DOI:** 10.3390/plants14131900

**Published:** 2025-06-20

**Authors:** Han Su, Xiaohu Jiang, Yanfen Liu, Zhuangyuan Cao, Ziqi Liu, Yuan Qin, Qing He, Hanyang Cai

**Affiliations:** 1 School of Future Technology, Haixia Institute of Science and Technology, Fujian Agriculture and Forestry University, Fuzhou 350002, China; suhan@fafu.edu.cn (H.S.); jiangxiaohu6754@163.com (X.J.); yanfen.liu@fafu.edu.cn (Y.L.); caozhuang09@163.com (Z.C.); lzq202303@163.com (Z.L.); yuanqin@fafu.edu.cn (Y.Q.); 2 Department of Biosciences, Durham University, Durham DH1 3LE, UK

**Keywords:** female germline, ERECTA, megaspore mother cell (MMC), integuments, functional megaspore (FM)

## Abstract

The female gametophyte is central to the reproductive success of flowering plants, with its development being tightly controlled by an intricate network of genes and signaling pathways. A deeper understanding of these regulatory mechanisms is essential for uncovering the complexities of plant growth and development. Recent studies have shed light on various aspects of female gametophyte development, highlighting the role of specific gene and signaling networks. Among these, the ERECTA family of leucine-rich repeat receptor-like kinase (RLK) in *Arabidopsis thaliana* has emerged as a key player, influencing multiple biological processes, particularly those governing reproductive development of the female gametophyte. This review focuses on the significant progress made in understanding the ERECTA family’s involvement in germline cell development, emphasizing its functional roles and signaling mechanisms in female gametophyte development.

## 1. Introduction

**Figure 1:**
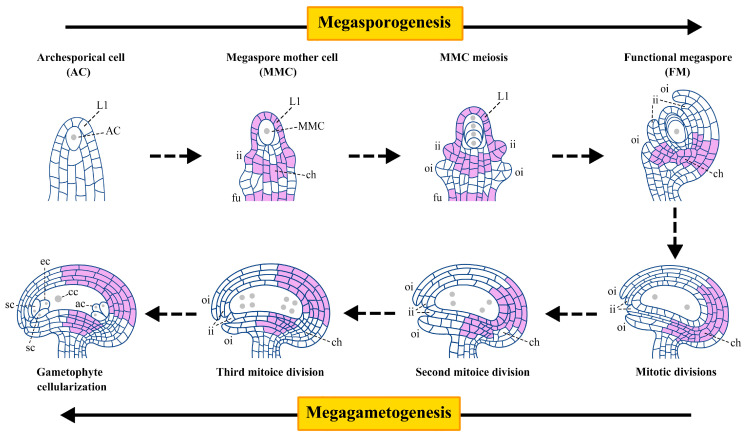
A scheme of female gametophyte development and the main expression domain of *ERECTA* in different stages in *Arabidopsis thaliana*. The plant female gametophyte development includes two stages: megasporogenesis and megagametogenesis. Megasporogenesis begins with the formation of an archesporial cell (AC) from a single subepidermal cell at the top of the ovule primordium and further differentiates into a megaspore mother cell (MMC). MMC undergoes meiosis to generate four megaspores. Among them, only one megaspore near the chalazal end successfully developed into a functional megaspore (FM), and the remaining three megaspores near the micropore end experienced degeneration. Megagametogenesis involves FM undergoing three mitoses to form the mature female gametophyte (FG), namely the embryo sac. The lilac region indicates the main expression position of *ERECTA* (*ER*) in ovules. In AC stage, *ER* is not expressed in ovules. In MMC stage, *ER* is expressed in the epidermal L1 layer cells, inner integuments, chalaza, and funiculus of the ovule primordia. From FM stage to FG stage, *ER* is distributed in the inner and outer integuments near the chalazal end. L1, nucellar epidermis cell layer; ii, inner integument; oi, outer integument; ch, chalaza; fu, funiculus; ac, antipodal cells; cc, central cell; sc, synergid cells; ec, egg cell.

Unlike animals, plants do not have a specific cell lineage (germline) to produce meiotic cells during early embryogenesis. In contrast, the female germline in plants is re-established from somatic cells in the reproductive organs of flowers, including the pistils [1]. In most sexually reproductive flowering plants, single sporophyte cells (somatic cells) usually undergo meiosis. The produced spores then develop into highly polarized and differentiated mature gametophytes which contain female gametes [1,2]. Female germline development in plants can be divided into two stages: megasporogenesis and megagametogenesis (Figure 1) [3]. Megasporogenesis begins with the formation of an archesporial cell (AC) from a single subepidermal cell in the distal nucellus. This cell elongates longitudinally and differentiates into a megaspore mother cell (MMC), which undergoes meiosis to generate four megaspores. Among them, only one megaspore becomes the functional megaspore (FM) near the chalazal end [3,4]. Megagametogenesis involves the formation of a syncytium through three mitotic divisions of FM, cellularization of the nucleus, and formation of a mature female gametophyte (FG, also known as an embryo sac) [3,5].

ERECTA (ER) encodes a leucine-rich repeat receptor-like kinase (LRR-RLK) in *Arabidopsis thaliana*. The ERECTA family (ERf) consists of ER and its functional paralogs, ERECTA-like 1 (ERL1) and ERL2. Based on the RLK family kinase domain and evolutionary analysis, ERf belongs to clade XIII of LRR-RLKs, which is characterized by an extracellular domain primarily composed of consecutive repeat LRRs [6,7,8]. The protein structure of ERfs is similar, composed of a signal peptide, cysteine region, LRR repeats, transmembrane domain, juxtamembrane domain, Ser/Thr kinase domain, and C-terminal tail. Of note, ER and ERL2 have 20 LRR repeats, while ERL1 contains 21 LRR repeats [7,8,9]. The similarity in protein structure reflects their common biological functions. Similar expression patterns indicate that they play similar or complementary functions at a specific time or under certain conditions. During different stages of ovule development in *Arabidopsis*, the expression position of ERf varies. In the AC stage, none of the *ER*f members are expressed in ovules. At the MMC stage, *ERL2* is expressed in the epidermal cells (L1 layer cells), inner integuments, and funiculus of the ovule primordia. *ER* is not only overlapped with *ERL2* but also expressed in the chalaza. Unlike *ERL2*, *ERL1* is expressed in the epidermal L1 layer cells and inner and outer integuments. These expression regions are similar across all three members. During the post-meiotic to FG stages, *ER* is distributed in the inner and outer integuments near the chalazal end. Different from *ER*, *ERL1* is expressed in the chalaza. *ERL2* exhibits weak expression in the integument and funiculus (Figure 1) [10,11,12].

*ER*f exhibits redundant roles in plant female germline development [10,13] loss-of-function mutants of *ER*f, specifically in *er-105 erl1-2 erl2-1* (simplified as *er erl1 erl2*) triple mutant, leading to female sterility in plants [13]. In the early stages of ovule development, *er erl1 erl2* mutants show abnormal ovule phenotypes at the MMC stage [11], while no disruption is observed in AC initiation or radial patterning [10]. In later stages of ovule development, both inner and outer integuments initiate normally in mutant ovules, but a decrease in integument cell proliferation leads to embryo sac exposure, which affects female gametophyte development and leads to complete sterility [10]. Therefore, *ER*f genes play critical roles in female gametophyte development and ovule integument growth during plant reproduction [14]. Here, this review focuses on the significant progress made in understanding the involvement of the ERECTA family in germline cell development, emphasizing its functional roles and signaling mechanisms in female gametophyte development.

## 2. ERf and Its Ligands EPFLs Mediate MMC Specification in Arabidopsis

Both ERf with the EPIDERMAL PATTERNING FACTOR (EPF)/EPF-like (EPFL) family are involved in MMC specialization and play a role in limiting the germline cell fate to a single MMC and promoting MMC differentiation. ERf receptors detected secreted cysteine-rich peptides from the EPF/EPFL family [15]. The *Arabidopsis EPF*/*EPFL*s have 11 members, with several genes shown to be involved in the *ER*f signaling pathway, interacting with *ER*f receptors [16,17,18,19]. Among them, single and double mutants of *EPFL1*, *EPFL2*, *EPFL4*, and *EPFL6* exhibited normal fertility, while triple mutants exhibited reduced fertility. Homozygous *epfl1/2/4/6* quadruple mutants were completely infertile, demonstrating that *EPFL1*, *EPFL2*, *EPFL4*, and *EPFL6* were required in a dose-dependent manner to mediate reproductive regulation. *EPFL1*, *EPFL4*, and *EPFL6* were predominantly expressed in the nucellus epidermal cells of ovule primordial [20], a pattern similar to that of *ER*f expression [11]. In *epfl1/2/4/6* mutants, two phenotypes were observed in ovules. The first phenotype involved excessively enlarged cells acquiring MMC cell identity, but these cells failed to develop into fully functional meiotic MMCs, indicating that *EPFL1*, *EPFL2*, *EPFL4*, and *EPFL6* redundantly limited germline cell fate to a single MMC without regulating its subsequent progression into meiosis. Additionally, a proportion of *epfl1/2/4/6* lacked MMC formation altogether [20].

ERf receptors are also involved in MMC specification. Two phenotypes were detected in *er erl1 erl2* ovules in *Arabidopsis*. The first phenotype exhibited ectopically enlarged MMC-like cells that failed to undergo subsequent meiotic division. The second phenotype showed an absence of MMC formation but instead promotes MMC differentiation [11]. MMC represents a transformation of cell fate in a subepidermal L2 layer cell at the apex of the ovule primordia. In *er erl1 erl2* mutants, the presence of multiple MMC-like cells could result from companion cells surrounding the MMC or from other somatic cells in the L2 layer aberrantly acquiring MMC fate. However, these MMC-like cells, despite their altered fate, fail to enter meiosis. The absence of MMC in some *er erl1 erl2* mutants may be due to the loss of *ER*f function, which normally mediates proper MMC initiation. In rice, the receptor-like kinase gene *OsERECTA2* (*OsER2*) is preferentially expressed during AC and MMC stages of ovule development and plays a critical role in female germline differentiation [21]. Single-cell RNA sequencing (scRNA-seq) of multiple single cells at different stages of female germline differentiation in *Arabidopsis* had revealed that the homologous gene *ERL1* of *OsER2* was a key gene in germline-related gene clusters and the epidermal nucellus cell subcluster [11]. These results collectively indicate that ERf with EPFLs are both involved in germline specification.

## 3. The EPFL-ERf and BR-BRI1 Signaling Modules Maintain the Correct Germline Progression by Activating the BZR1 Transcription Factor Family

### 3.1. EPFL-ERf and BR-BRI1 Signaling Activate BZR1-WRKY23/NSN1 to Restrict MMC Specialization

The EPFL-ERf signaling pathway is involved in the brassinosteroid (BR) transduction pathway, limiting only one MMC formation in the ovule through the expression of the target genes *WRKY23* transcription factor and nucleolar GTP-binding protein *NUCLEOSTEMIN-LIKE 1* (*NSN1*) activated by the *BRASSINAZOL-RESISTANT 1* (*BZR1*) transcription factor family but not directly involved in regulating meiosis. BZR1 family is a key component of the BR signaling pathway, a crucial regulator of plant growth and development [11,22]. Recent studies suggested that BZR1 might also play a central role in female germline fate determination. BR signaling is implicated in female germline cell development [20,23], with the BZR1 transcription factor family—including BZR1, BRI1-EMS-SUPPRESOR 1 (BES1), and their homologs BEH1, BEH2, BEH3, and BEH4—acting as key downstream regulators. BR perception is mediated by the receptor-like kinase BRASSINOSTEROID INSENSITIVE 1 (BRI1), which activates intracellular signaling cascades to regulate transcription [24]. In the context of MMC specialization, BR signaling transmits signals to the BZR1 family to regulate MMC fate [23]. Both the BRI1 and BZR1 transcription factor family are essential components of the BR signaling pathway and play a central role in MMC regulation [25]. A critical downstream target of *BZR1* is *WRKY23*, a BR-induced transcription factor specifically expressed in epidermal somatic cells surrounding the MMC. As a direct target of *BZR1*, *WRKY23* is transiently activated in response to BR signaling, ensuring only a single MMC identity to subepidermal cell at the distal end of the ovule primordium [23]. This highlights how BR-BRI1-BZR1 signaling orchestrates spatial regulation of female germline specification.

**Figure 2:**
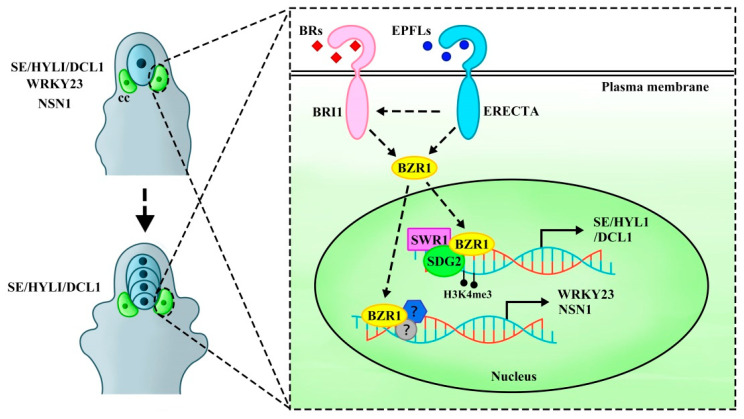
A scheme of ERECTA signaling regulates BRs transduction pathway to activate BZR1 transcription factor to inhibit companion cells fate transition. EPFLs-ERECTA signaling is involved in the regulation of the BRs-BRI1 signal transduction pathway to activate the BZR1 transcription factor family. By dephosphorylating BZR1 into the nucleus and instantaneously activating *WRKY23* and *NSN1* expression, companion cells are inhibited from acquiring MMC fate. This results in only one MMC but does not regulate entry into meiosis. SWR1 and H3K4me3 histone modification SDG2 activates BZR1 to promote the deposition of H2A.Z at downstream target genes *SE*, *HYL1*, and *DCL1* to activate the expression of these genes. This regulation ensures that only a single cell in the ovule primordium acquires the MMC fate and limits only one MMC to enter the decelerating division to form an FM. cc, companion cells.

Genetic analysis had shown that *bri1-116* and *qui-1* (*bes1-1 bzr1-1 beh1-1 beh3-1 beh4-1* quintuple mutants) [23] had multiple MMC phenotypes, similar to those observed in *er erl1 erl2* and *epfl1/2/4/6* mutants [20]. Notably, transcriptional and expression levels of the *BRI1* and *BZR1* family were significantly reduced in *er erl1 erl2* and *epfl1/2/4/6* mutants. Introducing the *BES1* and *BZR1* gain-of-function mutants (*bes1*-*D* and *bzr1*-*1D*) into *er erl1 erl2* and *epfl1/2/4/6* mutants partially rescued the multiple MMC phenotypes and fertility, indicating that BRI1-BZR1 signal acted downstream of EPFL-ERf signal. Furthermore, compared with *er-105* or *bri1-119* mutants, the *er-105 bri1-119* double mutants were completely sterile, showing more severe defects. MMC-like cells were significantly increased in *er-105 bri1-119* mutants, suggesting a genetic interaction in preventing excessive MMC-like cell formation. In addition, the EPFL-ERf signaling pathway restricts the differentiation of multiple MMCs by activating NSN1, a direct target of the BZR1 family, and the binding between BZR1 and NSN1 depends on the EPFL-ERf complex [20]. Consistent with this, *WRKY23* transcriptional levels were significantly reduced in *er erl1 erl2*, *epfl1/2/4/6*, and *er-105 bri1-119* mutants. The multiple MMCs phenotype in *nsn1 wrky23* double mutants was more severe than in either single mutant, suggesting that *NSN1* and *WRKY23* genetically interacted to restrict MMC specialization [20]. Combining these, the EPFLs-ERf signal regulates the BR-BRI1-BZR1 signaling pathway and inhibits MMC fate by activating the expression of *BZR1* downstream target genes *NSN1* and *WRKY23*. These findings suggest that the EPFLs-ERf signal is interconnected with the BR hormone signaling pathway, revealing a gene regulatory network (GRN) that regulates MMC specialization (Figure 2).

### 3.2. The SWR1-SDG2-ER Module Activates BZR1 to Promote H2A.Z Deposition and Limit Germline Specialization

*ER* interacts with epigenetic factors, such as the nucleus-localized ATP-dependent chromatin remodeling complexes *SWI2*/*SNF2-RELATED 1* (*SWR1*) and *SET DOMAIN GROUP 2* (*SDG2*), to promote the H2A.Z histone variants deposition on the promoters of *HYPONASTIC LEAVES 1* (*HYL1*), *DICER-LIKE 1* (*DCL1*), and *SERRATE* (*SE*) by activating the BZR1 transcription factor family, changing chromatin status and activating gene expression. In addition to limiting germline cell fate, the SWR1-SDG2-ER module can also restrict only a single MMC to enter meiosis. *ER* was reported to interact with *SWR1* and *SDG2* to participate in female gametophyte development [26,27]. SWR1 is involved in various developmental processes, including leaf shape regulation, organ size, meiosis, and germline specialization [26,28,29,30,31]. It promotes the incorporation of H2A.Z histone variants into nucleosomes and catalyzes the replacement of H2A-H2B dimers with H2A.Z-H2B dimers [32]. The deposition of H2A.Z alters histone–DNA interactions, chromatin structure, and transcription factor accessibility, thereby affecting nucleosome stability and gene transcription regulation [33,34,35,36]. Histone modifications, such as H3 lysine 4 trimethylation (H3K4me3) and histone H3 lysine 27 trimethylation (H3K27me3), are key markers of transcriptional activity and repression. H3K4me3 serves as an active transcriptional marker [37], while H3K27me3 functions as a transcriptional inhibitory marker [38]. H2A.Z is preferentially associated with H3K4me3 at promoters and H3K27me3 at enhancers [34]. SDG2 is involved in H3K4 methylation, and its loss of function leads to significant defects in sporophyte and gametophyte development [39]. Research demonstrated that the SWR1-ER signaling pathway regulated inflorescence structure by promoting the enrichment of H2A.Z and SDG2-mediated H3K4me3 modifications on auxin-associated genes. *SDG2* activates the expression of *PACLOBUTRAZOL RESISTANCE* (*PRE*s) genes via H3K4me3 modification, a process regulated by the SWR1-ER signaling pathway. This highlights an interaction between the SDG2 and SWR1-ER signal in inflorescence development [40].

Moreover, ACTIN-RELATED PROTEIN 6 (ARP6), a subunit of the SWR1 complex [41,42], had been investigated in a study that utilized ethyl methane sulfonate (EMS) to screen for enhancers in *arp6* mutants. This screening isolated the *aeh1* enhancer mutants that significantly enhanced the phenotypic defects of *arp6* mutants [43]. *aeh1* mutant was phenotypically similar to *er-105* mutant in terms of inflorescence and silique development [7,13], though neither exhibited fertility defects [10]. Therefore, *aeh1* (named *er-119*) was a point mutant in the *ER* gene that resulted in premature termination of translation [43]. The study further indicated that the extra MMC-like cells in *arp6 er-119 sdg2* triple-mutant ovules acquired MMC identity and subsequently underwent meiosis to generate multiple FMs with FM characteristics. Together, these findings suggest that SWR1-SDG2-ER form an epigenetic module that limits the fate of only one subepidermal cell in the ovule primordia to become the MMC, thereby regulating the progression into meiosis. In *arp6 er-119 sdg2* mutants, two populations of FMs were observed: one where multiple FMs formed along the distal–proximal axis (Type Ⅰ), indicating that these may have arisen from a single MMC division without programmed cell death (PCD), and another where multiple FMs were arranged side by side on the distal–proximal axis (Type Ⅱ), suggesting that they likely originated from different MMCs [27]. The abnormal ovule number of *arp6 er-119 sdg2* triple mutant is significantly higher than that of single and double mutants, further supporting the role of the SWR1-SDG2-ER signaling module in controlling ovule development [27]. Therefore, the SWR1-SDG2-ER signaling module restricts MMC fate to a single cell in the ovule primordia and promotes its subsequent meiosis by activating downstream targets such as the BZR1 transcription factor family [27]. Although BZR1-WRKY23/NSN1 can restrict MMC specification, the phenotype of multiple MMC-like cells in *qui-1* mutant ovules is far more severe than in *wrky23* and *nsn1* mutants, and some of the multiple MMCs in *qui-1* mutants can enter meiosis—a phenotype absent in *wrky23* and *nsn1* mutants [23]. This suggests that the BZR1 transcription factor family may regulate additional target genes to restrict the entry of only one MMC into meiosis. Additionally, *HYL1*, *DCL1*, and *SE*—*BZR1* target genes—are critical factors in megagametogenesis of FM development into embryo sacs and miRNA processing [44]. *BZR1* directly activates *HYL1*, *DCL1*, and *SE* by binding to their promoters, a process regulated by the SWR1-SDG2-ER signaling module. In both *arp6 er-119 sdg2* mutants and a *BZR1* knock-out mutant *sex-1*, a significant decrease in H2A.Z deposition was observed at the −1 to +1 nucleosome positions near the transcription start sites TSS of *HYL1*, *DCL1*, and *SE*, indicating that the SWR1-SDG2-ER signaling module, along with the BZR1 transcription factor family, promoted H2A.Z deposition at *HYL1*, *DCL1*, and *SE* genes to activate their expression [27]. Moreover, *er-119 hyl1* double-mutant ovules exhibited multiple MMCs capable of meiosis, and these ovules contained two additional populations of FMs. No obvious phenotypes of multiple MMC-like cells were detected in *hyl1*, *dcl1*, or *se* mutants, likely due to functional redundancy between these genes. This indicated that *ER* and *HYL1* genetically interacted to specifically limit the formation of a single MMC and subsequent meiosis [27]. However, the multiple FMs phenotype was not observed in *bri1-116* [23], suggesting that the BZR1 transcription factor family might regulate germline cell fate through an ERf-related signaling pathway independent of BR signaling. In conclusion, plasma membrane-localized BRI1 and ERf regulate the germline fate by activating the activity of the BZR1 family, providing new insights into the precise control of germline establishment and development (Figure 2).

## 4. ER Participates in Epigenetic Pathways to Regulate Female Gametophyte Development

Epigenetic pathways play an important role in female gametophyte development. Recent studies have shown that in addition to interacting with ER and SDG2, SWR1 also interacts with ER and its downstream mitogen-activated protein kinase (ER-MPK) signaling pathway to regulate female gametophyte development [44]. Compared to *arp6* and *er-119* single mutants, *arp6 er-119* double mutants exhibited a substantial increase in poorly developed ovules and abnormal female gametophytes. The introduction of a complementary vector *pER:ER*, carrying the *ER* gene sequence, restored the reduced seed-setting rate and female gametophyte defects in *arp6 er-119* double mutants to a level comparable to *arp6* mutant, confirming that the enhanced female gametophyte defects in *arp6 er-119* were caused by *er-119* mutation [44]. *MIR398c* is the only miRNA gene with significantly changed expression in RNA-Seq analysis of WT, *arp6*, *er-105*, and *arp6 er-119* ovules [43]. The expression level of *MIR398c* increased in *arp6 er-119*, *arp6 mpk6*, and *er-105 mpk6* ovules. Additionally, the occupancy rate of RNA Polymerase Ⅱ (Pol Ⅱ) on the *MIR398c* gene body increased, along with an increase in the active transcription marker H3K4me3, and a decrease in the inhibitory marker H3K27me3. These findings indicated that *MIR398c* was actively transcribed in these double mutants, which was consistent with the results of *MIR398c* overexpression [44]. However, *MIR398c* transcription activation was observed only in double mutants mentioned above, not in *arp6* alone, suggesting that while SWR1 helped inhibit ER-MPK-regulated *MIR398c* gene transcription, changes in nucleosome status controlled by SWR1 were insufficient on their own to activate *MIR398c* transcription. Therefore, SWR1 and ER-MPK6 jointly inhibit MIR398c expression in ovules [44].

**Figure 3:**
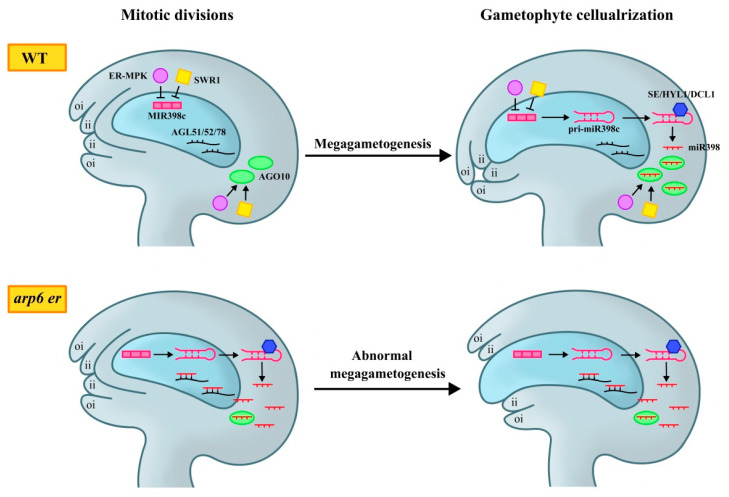
A scheme of the temporal and spatial control of miR398 biogenesis by ER-MPK and SWR1 to ensure female gametophyte development and proper ovule morphogenesis. In WT ovules, ER-MPK and SWR1 inhibit the transcription of *MIR398c* at the early stage of megagametogenesis. In the mature stage of the ovule, *MIR398c* is transcribed into pri-miR398c in female gametophytes. Subsequently, pri-miR398c is transferred from the female gametophyte to the sporophyte tissue, where it is processed into mature miR398 by *SE*, *HYL1*, and *DCL1*. ER-MPK and SWR1 promote the expression of AGO10 at the chalaza. AGO10 can effectively prevent miR398 from entering female gametophytes and binding to its target genes *AGL51*/*52*/*78*, thus ensuring the normal expression of *AGL*s in female gametophytes and further maintaining female gametophyte development and integument growth. In *arp6 er* mutant ovules, *MIR398c* is over-accumulated in female gametophytes and pri-miR398c is transcribed in advance. Due to the reduction of AGO10, miR398 cannot be captured, resulting in the ectopic expression of miR398 in female gametophytes to inhibit the expression of *AGL*s. As a result, these mutant ovules lead to abnormal development of female gametophytes and prominent embryo sacs.

Furthermore, ensuring the normal function of miR398 target gene *AGAMOUS-LIKE* (*AGL*) *51/52/78* in female gametophytes is necessary for maintaining normal ovule morphogenesis. In ARP6 and ER-MPK pathway mutants, the accumulation of miR398 leads to abnormal female gametophyte development by inhibiting the expression of its three functionally redundant candidate target genes: *AGL51*, *AGL52*, and *AGL78*, which belong to the Mb subclade of the MADS-box gene family [45]. These genes are specifically expressed in female gametophytes. The ovule phenotype in *agl78* resembles those in *arp6 er-119*, *arp6 mpk6*, *er-105 mpk6* mutants, and *MIR398c* overexpression, suggesting that *AGL78* plays a predominant role in gametophyte development. When *AGL78* was introduced as an anti-miR398 construct driven by a natural promoter with five mutation sites into *arp6 er-119* double mutants, the seed setting rate and fertility improved significantly compared to *arp6 er-119*, and the proportion of abnormal ovules was significantly reduced. *AGL78* overexpression partially rescues the low seed-setting rate and female gametophyte defects in *arp6 er-119*. These findings demonstrate that excessive accumulation of miR398 in ARP6 and ER-MPK pathway mutants suppresses the expression of *AGL51/52/78*, thereby disrupting normal female gametophyte development (Figure 3) [44].

Furthermore, *MIR398c* is preferentially expressed in mature WT female gametophytes while *ARP6*, *ER*, and *MPK6* are widely expressed in ovules, including female gametophytes and sporophyte tissues. *arp6 er-119*, *arp6 mpk6*, and *er-105 mpk6* mutants caused a higher *MIR398c* expression level in immature female gametophytes compared to WT, indicating that SWR1 and ER-MPK6 acted to suppress *MIR398c* expression during the early stages of female gametophyte development. The *pri-miR398c* transcript is produced within mature female gametophytes and is subsequently transferred from female gametophytes to surrounding sporophyte tissue, where it is processed by *HYL1*, *DCL1*, and *SE* [44].

However, during mitosis to mature female gametophytes, ARGONAUTE10 (AGO10) enriched at chalazal isolates miR398 here, preventing mature miR398 from moving to female gametophytes. miR398 is identified as the second-most abundant miRNA in the immunoprecipitation of AGO10 [46], which has been reported to reduce miR165/6 activity and promote its degradation by isolating miRNAs [46,47]. AGO10 transcripts were significantly reduced in *arp6 er-119*, *er-105 mpk6*, and *arp6 mpk6* mutants. *ago10-12* mutant, which did not show obvious defects in female gametophyte development [48], enhanced the development defects in *arp6*, *er-105*, and *arp6 er-105* ovules. This suggests that SWR1 and ER-MPK regulate *MIR398c* in the mature female gametophyte by promoting AGO10 expression in the chalaza, thereby spatially isolating *MIR398c* and inhibiting its transcription to activate *AGL51/52/78* expression during female gametophyte development (Figure 3) [44].

These findings highlight the role of AGO10 in miR398 regulation, which is activated by SWR1 and ER-MPK pathways, thus providing new insights into the movement of small RNAs and the communication between different plant tissues during development [44]. Furthermore, the coordinated development of sporophyte and gametophyte tissues is essential for ovule formation and fertilization. The MPK6 cascade and ER regulate ovule morphology similarly, both of which promote female gametophyte development. ER-MPK cascade inhibits *MIR398c* transcription and miR398 accumulation in female gametophytes via a gatekeeping mechanism. Thus, the ER-MPK cascade ensures the proper function of miR398 target genes *AGL51/52/78* in the female gametophyte and regulates communication between female gametophytes and surrounding sporophyte tissues, ultimately ensuring normal ovule morphogenesis [44].

## 5. ERf Signaling Pathway Is Involved in Integument Development

The development of plant ovules encompasses several key processes, including primordium formation, megasporogenesis, megagametogenesis, and integument development [12]. As sporogenous tissues, the integuments gradually enclose the nucellus during megasporogenesis and megagametogenesis, providing protection and nourishment to the embryo sac. In *Arabidopsis*, the inner and outer integuments are composed of two epidermal cell layers in the early stages of ovule development. As development progresses, the inner integuments grow to consist of three cell layers [49]. These cell layers expand through anticlinal cell division and progressively surround the nucellus, the site of megasporogenesis and embryo sac development [50,51,52]. Defects in embryo sac formation may arise from integument defects, as the gametophyte cannot develop properly if the nucellus is not enclosed [53]. Megagametogenesis must be coordinated with the surrounding sporophyte integument [54,55], which is considered to be responsible for establishing polarity in the female gametophytes [55,56].

ERf is expressed in the integument and plays a crucial role in ovule development and fertility by regulating cell proliferation in the integument, which is essential for normal integument growth. *er erl1 erl2* triple mutants exhibited significant defects in integument growth, unlike *er-105* or *erl1-2 erl2-1* mutants. The *er erl1 erl2* triple mutants showed reduced cell division, leading to short integuments that failed to enclose the nucellus. Consequently, the embryo sac is exposed and undergoes abnormal development, leading to abortion and the formation of disorganized small cell clusters. In comparison to WT, the cells in the outer integuments of *er erl1 erl2* mutants became increasingly disordered starting from stage 3-Ⅰ, eventually arresting at the two-nucleate embryo sac stage [10]. *PRETTY FEW SEEDS 2* (*PFS2*), also known as *WOX6*, is a *WUSCHEL* (*WUS*)-related homeobox gene which is particularly strongly expressed during the initiation and growth of the inner integuments [10,57,58]. *PFS2* is proposed to control ovule patterns by regulating the time of cell differentiation [57]. Interestingly, the loss-of-function mutant of *PFS2* promoted the further development in *er erl1 erl2* mutant ovules beyond the two-nucleate stage, indicating that loss of *PFS2* function partially rescued the ovule defects in *er erl1 erl2* mutants [10]. This indicated that *ER*f genetically interacted with *PFS2* to jointly regulate integument cell proliferation and gametocyte differentiation. Notably, the expression domains of *ER* and *ERL1* in *pfs2-1* mutants remained unchanged, and the expression domain of *PFS2* did not alter in *er erl1 erl2* triple mutants. However, the expression level of *PFS2* increased in *er erl1 erl2* mutants, suggesting that while *ER*f did not spatially restrict *PFS2* expression, it may have inhibited the expression level of *PFS2*. The elevated PFS2 expression in *er erl1 erl2* ovules could contribute to gametophyte abortion in these mutants [10]. Furthermore, the *pfs2-1* mutation affects the *ER*f mutant phenotype, indicating that *pfs2-1* mutation exerts an epistatic effect within this genetic pathway. This demonstrates that ERf and PFS2 promote integument growth through distinct but interconnected pathways.

### 5.1. SDF2-ERdj3-BiP Chaperone Complex Mediates ERf Translocation from Endoplasmic Reticulum to Plasma Membrane to Maintain Normal Integument Growth

*Arabidopsis* endoplasmic reticulum-localized DnaJ family 3B (ERdj3B) has a crucial function in integument development by regulating the translocation of the ERf receptor kinase [59]. In mammalian and plant cells, the endoplasmic reticulum-localized co-chaperone DnaJ (Hsp40), such as ERdj3, directly binds to immunoglobulin heavy chain-binding proteins (BiPs) to activate them. The BiPs further bind to stromal cell-derived factor 2 (SDF2) to form the SDF2-ERdj3-BiP chaperone complex, which participates in protein folding, translocation, and quality control [60,61,62]. Mutation of ERdj3B in the *Arabidopsis* Landsberg *erecta* (L*er*) ecotype leads to inner integument defects at stage 3-Ⅲ, and later, the inner integuments of *erdj3b-5*^L*er* cas9^ mutant ovules fail to fully cover the embryo sac morphology and abortion during stages 3-Ⅴ to 3-Ⅵ. However, this mutation does not affect the outer integument development [59].

**Figure 4:**
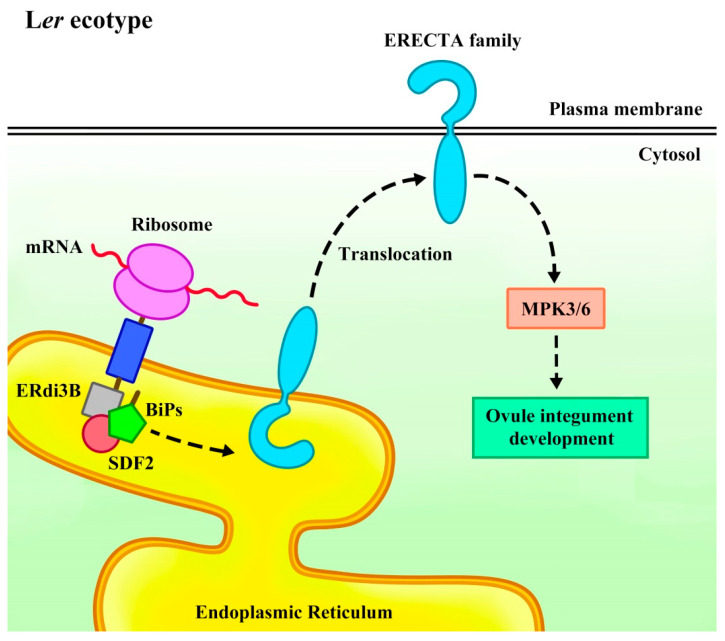
A scheme of the SDF2-ERdj3B-BiP complex regulates the ERECTA family from the endoplasmic reticulum to the plasma membrane translocation model. In the *Arabidopsis* Landsberg *erecta* (L*er*) ecotype, the SDF2-ERdj3B-BiP complex binds to nascent peptides and promotes their folding, thereby mediating the ERECTA family translocation from the endoplasmic reticulum to the plasma membrane (PM). They then activate the downstream MPK3/6 cascade to regulate ovule integument growth. In *erdj3b er* or *sdf2 er* mutants, the absence of ERdj3B or SDF2 inhibits the translocation of the ERECTA family. Meanwhile, the distribution of ERL1 and ERL2 in PM decreases, which is not enough to maintain the normal development of ovule integument, resulting in integument development defects and ovule abortion.

Through map-based cloning, the *ER* gene was identified as a natural modifier of *ERdj3B*. In *erdj3b* mutant plants, the protein abundance and plasma membrane distribution of ERf were significantly reduced. The *erdj3b er* double mutants displayed significant defects in the inner integuments under heat stress, while additional mutations in *ERL1* or *ERL2* exacerbated the integument defects, resulting in abnormalities in both the inner and outer integuments and leading to ovule abortion. These results indicated that *ERdj3B* genetically interacted with *ER*f to coordinate plant development, and *erdj3b* mutations enhanced the ovule abortion phenotype in *er erl1 erl2* triple mutants. Similarly, *er sdf2* double mutants exhibited similar defects in the inner integuments, confirming that SDF2 was also crucial for normal integument development that depends on ER [59]. Moreover, mutations in mitogen-activated protein kinase 3 (MPK3) and MPK6 lead to abnormal growth and development of the inner and outer integuments, with cell division arrest in later stages [63]. The MAPK signaling cascade is a three-layer kinase module [64] which plays a crucial role in regulating both stress responses and plant growth and development [65,66,67]. MPK3 and MPK6 promote integument cell division, and *mpk3 mpk6* double mutants are female sterile due to sporophyte defects [63]. The MPK3/MPK6 cascade redundantly acts downstream of ERf in multiple developmental processes, including ovule development [17,59,68,69,70,71,72]. It is therefore proposed that the SDF2-ERdj3B-BiP chaperone complex mediates the translocation of ERf receptor kinase from the endoplasmic reticulum to the plasma membrane, activating the downstream MPK3/MPK6 cascade and regulating intercellular interactions during integument development to maintain normal integument growth (Figure 4) [59,63]. These findings reveal that molecular chaperones play a critical role in the ERf signaling pathway.

### 5.2. EPFL-ERf-SERK Signaling Regulates Integument Development

Recent studies also showed that the leucine-rich repeat RLK family, *SOMATIC EMBRYOGENESIS RECEPTORLIKE KINASES* (*SERK*s), overlapped in expression with *ER*f during ovule integument development in *Arabidopsis*. SERKs were detected in both the inner and outer integuments. The *serk1/2/3* triple mutants showed integument retardation, abnormal embryo sacs, and female sterility, similar to *er erl1 erl2* triple mutants [10,12]. The higher-order *serk1*/*2*/*3 er erl1 erl2* mutant ovules exhibited extremely short integuments, resembling the phenotypes seen in both *er erl1 erl2* and *serk1*/*2*/*3* mutants. However, no significant differences in the cell number or length of outer integuments were observed between *serk1/2/3*, *er erl1 erl2*, and *serk1/2/3 er erl1 erl2* mutants, indicating that *SERK*s and *ER*f coordinated integument development in the same pathway [12]. *EPFL1* to *6* are expressed in epidermal cells of the ovule development. The *epfl1/2/3/4/5/6* sextuple mutants showed integument defects similar to those seen in *er erl1 erl2* and *serk1/2/3* mutants, with *EPFL4/6* enhancing the interaction between *ER* and *SERK*s [12]. SERK3, as a co-receptor of BRI1 and receptor-like protein kinases GassHO1/2 (GSO1/2), regulates BR signal and embryonic cuticle development by sensing BR and TWISTED SEED1 (TWS1) peptides [73,74]. Application of exogenous BR or TWS1 significantly enhanced the interaction between *SERK3* and *BRI1* or *GSO2* [73,74] but not with *ER* [12]. Interestingly, EPFL6 treatment did not enhance the interaction between *SERK3* and *GSO2* or *BRI1*, suggesting that the ER-SERK receptor complex was specifically induced by EPFL1-to-6 ligands to control integument morphogenesis [12]. Although BR signal was involved in regulating outer integument development, its effects were relatively mild compared to the severe integument defects observed in *epfl1/2/3/4/5/6*, *er erl1 erl2*, and *serk1/2/3* mutants [75]. This demonstrates that the EPFL-ER-SERK signaling plays a more fundamental regulatory role in integument development, independent of the BR signaling pathway. In summary, these phenomena reveal the complexity of integument development and highlight the importance of the synergy between distinct signaling pathways in plant reproductive development.

## 6. Conclusions and Future Perspectives

Recent studies have made major advances in understanding the role of ERf in regulating ovule development and related processes. ERf participates in different pathways to coordinately regulate MMC development, female gametophyte formation, and integument growth. ERf is mainly expressed in the L1 layer cells of ovule primordia and integument cells during ovule development. They can restrict germline cell fate transition to a single MMC and promote MMC differentiation during ovule development while simultaneously sustaining normal female gametophyte development by regulating cell proliferation in integument [10,11]. The EPFL-ERf and BR-BRI1signaling pathway are involved in BR transduction pathway, limiting the formation of only one MMC in ovules by activating the target genes *WRKY23* and *NSN1* expression through *BZR1* family, without directly regulating meiotic [20] or by activating the BZR1 family through the SWR1-SDG2-ER module to promote H2A.Z deposition on *SE*, *HYL1*, and *DCL1* promoters. This interaction not only restricts germline cell fate but also ensures that only a single MMC enters meiosis [27]. These findings demonstrate that ERf participates in multiple signaling pathways to precisely regulate plant reproductive development through coordinated regulation of BZR1 family activity (Figure 2). Furthermore, the SWR1 and ER-MPK signaling pathway inhibit *MIR398c* transcription in female gametophytes and activate *AGO10* expression in chalazal. AGO10 inhibits the mature miR398 accumulation in female gametophytes, thereby maintaining the expression levels of the specific genes *AGL51*/*52*/*78* to ensure normal female gametophyte development (Figure 3) [44]. In ovule integument development, SDF2-ERdj3B-BiP chaperone complex mediates ERf translocation from the endoplasmic reticulum to the plasma membrane in *Arabidopsis* L*er* ecotype, likely sustaining normal integument growth through the MPK3/MPK6 cascade (Figure 4) [59]. Additionally, the EPFL-ER-SERK signal is involved in regulating normal integument morphologenesis [12]. Research on ERf has provided critical insights into the molecular mechanisms governing plant reproductive development. By dissecting the synergy between ERf and diverse signaling pathways, we can gain deeper insights into how plants regulate differentiation and germline cell development through complex signaling networks. These studies not only elucidate fundamental principles of plant reproductive development but also provide a theoretical basis for crop breeding and genetic improvement.

Despite these advances, the mechanism behind the absence of MMC formation in *er erl1 erl2* mutant ovules [11,20] remains unknown. Previous studies had shown that mutations in *SPOROCYTELESS/NOZZLE* (*SPL/NZZ*) and *WUS* resulted in the loss of female germline formation due to the absence of MMC. *WUS* expression decreased in *spl/nzz* mutants, indicating that *WUS* acted downstream of *SPL/NZZ* during MMC development [76,77]. *WUS* regulated MMC formation by indirectly activating the expression of two redundant genes, *WINDHOSE 1* (*WIH1*) and *WIH2*. Mutations in *TORNADO 2* (*TRN2*)/*EKEKO* and *TORNADO*/ *LOPPED 1* (*TRN1*/ *LOP1*) exhibited similar phenotypes to *wih1 wih2* double mutants, both of which led to organ distortion and the absence of MMC in ovules. This suggests that *SPL/NZZ*, *WUS*, *WIH1/2*, and *TRN1/2* may function in the same pathway [77]. The absence of MMC formation in *er erl1 erl2* and *epfl1/2/4/6* mutant ovules resembles the phenotypes of *spl/nzz*, *wus*, *wih1 wih2*, and *trn1/2* mutants, suggesting that the EPFL-ER signaling pathway may be involved in this pathway to promote MMC formation. In addition, WUS is necessary for integument initiation, as its absence results in the inability to form integuments, leading to the arrest of embryo sac development [78]. *TRN1* and *TRN2* are predominantly expressed in the nucellus and ovule integuments [77], which overlap with the expression domain of ERf. It is speculated that the deletion of *TRN1* and *TRN2* may lead to similar integument defects. Therefore, the EPFL-ER signal may be involved in the regulation of ovule integument development in association with WUS, TRN1, and TRN2.

Recent studies had suggested that both full-length ER protein and its N-terminal truncated ER kinase domain (ER^KD^) were detected in the nucleus of *Arabidopsis* root. ER underwent endocytosis to migrate the truncated ER^KD^ into the nucleus, where it interacted with *SWI3B* subunit of SWI/SNF chromatin remodeling complexes (CRCs), implying that ER^KD^ might have a ligand-independent non-canonical signaling function. The kinase domains of all ERf proteins contain a functional nuclear localization signal (NLS) and leucine-rich nuclear export signal (NES) [79]. This finding implies that the kinase structure of ER family proteins may translocate to the nucleus and interact with nuclear localization genes related to reproductive development, such as *SPL*, *WUS*, and *BZR1*, to regulate male and female gametophyte development. These findings yield fresh perspectives on the role of ERf in regulating reproductive development in *Arabidopsis*.

In the process of ovule development, close signaling exchanges occur between sporophyte and gametophyte tissue, ensuring their coordinated development. This system offers an excellent model for investigating the fundamental mechanisms controlling germline formation, cell growth and division, and gamete cell fate specification [4]. In Arabidopsis, female gametophyte development involves precise regulatory mechanisms at multiple stages, encompassing both signal transduction and non-cell-autonomous signaling transduction, such as transcription factors, phytohormones, miRNAs and epigenetic patterns. As technology advances and precision techniques continue to develop, new perspectives have been provided for a deeper understanding of the molecular mechanisms underlying female gametophyte development in plants. Techniques such as fluorescence-activated cell sorting (FACS) with single-nucleus Hi-C (snHi-C) [80] can map the three-dimensional chromatin structure in different cell types of female gametophytes, revealing their dynamic changes during developmental progression. High-resolution microscopy techniques, such as confocal laser scanning microscopy (CLSM) and transmission electron microscopy (TEM) [81], provide crucial support for the precise study of the cell morphology and subcellular structures of female gametophytes. Additionally, the effects of environmental changes on plant female gametophyte development—such as temperature, photoperiod, and other environmental factors—reveal the interplay between environmental conditions and physiological mechanisms, providing valuable insights for improving reproductive capacity and yield in plants under adverse environmental conditions.
